# Substance Use and Psychosocial Status among People Living with HIV/AIDS Who Encountered HIV Stigma in China: Stratified Analyses by Socio-Economic Status

**DOI:** 10.1371/journal.pone.0165624

**Published:** 2016-11-08

**Authors:** Chen Zhang, Xiaoming Li, Yu Liu, Shan Qiao, Yuejiao Zhou, Zhiyong Shen, Yi Chen

**Affiliations:** 1 Divison of Epidemiology, Vanderbilt University, Nashville, Tennessee, United States of America; 2 Department of Health Promotion, Education, and Behavior, Arnold School of Public Health, University of South Carolina, Columbia, South Carolina, United States of America; 3 Department of HIV/STD Prevention, Guangxi CDC, Nanning, Guangxi, China; National Center for AIDS/STD Control and Prevention, China CDC, CHINA

## Abstract

This study examined whether the impact of HIV stigma on psychosocial status and substance use among people living with HIV/AIDS (PLWHA) differed by their socio-economic status (SES) in a Chinese setting. A total of 2,987 PLWHA were recruited from 12 sites with the highest number of cumulative HIV incidence in Guangxi, China. Participants were asked to provide information regarding their psychosocial status (e.g., depression, anxiety), history of substance use (e.g., tobacco, alcohol and drug) and SES (e.g., education, monthly income, residence type, and job category). By employing stratified multivariate regression analyses, we assessed stratum-specific impacts of HIV stigma on PLWHA’s psychosocial status and behaviors of substance use based upon participants’ SES. The impact of HIV stigma differed significantly on psychosocial status across SES gradients. Psychosocial status among people with higher education was more sensitive to HIV stigma compared with PLWHA who were less educated. The odds of substance use behaviors were higher among people with better monthly income than their low-income peers. Our study is the first paper to document the paucity of SES stratified analyses between HIV stigma and psychosocial status and substance use among PLWHA in China. We call for tailored intervention programs to target PLWHA with different backgrounds and characteristics in order to help them to better manage their seropositivity.

## Introduction

By the year of 2016, it is estimated that a total of 37 million people living with HIV/AIDS (PLWHA) worldwide [1,2]. With the advancement and availability of HARRT, HIV/AIDS has transited from a lethal to a manageable chronic disease[1,2]. However, the prognosis differed significantly based upon PLWHA’s socio-economic status (SES) [3,4]. SES is a multi-dimensional and complex measure that has been defined variously in history, from a measurable to an abstract construct that reflects one’s access to collectively desired resources [5]. In the health related studies, SES is usually measured by educational attainment, occupation, and income levels [6].

Existing studies have indicated that low SES may be associated with higher risk of substance use [7], and the association with mental health was inconclusive [8,9]. On the other hand, studies have indicated the PLWHA with higher SES would have better prognosis as they usually possessed more intangible (e.g., social support, access to health care) and tangible resources (e.g., medication, food supplies, housing) compared to their peers who were at lower SES [10–13].

In addition to PLWHA’s prognosis, studies have highlighted the importance of addressing PLWHA’s psychosocial needs and behaviors of substance use, as those with psychosocial comorbidities and problems of substance use are at a higher risk of sexual disinhibition, not adhering to ART, having lower quality of life and encountering other health problems, which might negatively influence the social and individual well-beings [14,15]. In addition to their psychosocial distress, stigmatized experience is commonly encountered in the life context of PLWHA [16,17].

Stigma was defined as a discrediting attribute of a given individual as a result of the possession of socially devalued marks[18]. In a continuous social process and complex social interactions including labeling, stereotyping, separation, status loss, and discrimination, the possessor of the socially devalued marks has been stigmatized [18,19]. Following a similar process, HIV stigma against PLWHA has been developed and maintained within different social and cultural contexts [15,20,21]. Scholars have revealed a continuum of devastating consequences (physical, emotional and financial burdens) of HIV stigma against PLWHA in China and other settings [16,17,22–24].

In the current study, we aimed to examine SES stratum specific associations between different types of HIV stigma and psychosocial well-being as well as behaviors of substance use among a group of PLWHA recruited from Chinese settings. Our hypotheses are that HIV stigma is positively associated with psychological distress and problems of substance use among PLWHA, and such impacts may interact with different SES gradients.

## Methods

### Study site

The current study was conducted in Guangxi Zhuang Autonomous Region (Guangxi) in China. Guangxi is located in the Southwest of China with a total population of 50 million with 32% of Zhuang-ethnic and 8% of other minorities[25]. HIV epidemic has been surging in Guangxi. By the end of 2014, Guangxi has ranked first among all 31 provinces in terms of cumulative HIV seropositive cases[26,27].

### Study design

Details of the study protocol and the pre-established sampling scheme were documented elsewhere [16,17]. Briefly, from 2012 to 2013, we collaborated with Guangxi Center for Disease Control and Prevention (Guangxi CDC) to target a total of 29,606 HIV/AIDS cases from the 12 sites in Guangxi, including two cities and ten counties with the highest number of cumulative HIV incidence. We randomly selected approximately 10% of cases from the sampling pool and finally recruited 3,002 HIV patients in the study, with about 10% refusal rate. Among the 3,002 patients, 2,987 (99.5%) of them completed the cross-sectional survey and were included in the current analysis. Specifically about 80% of the included participants completed the questionnaire assisted by the well-trained local CDC staff and health care workers. The rest of the sample completed the questionnaire on their own. Written consent was obtained from all participants. The Institutional Review Boards at Wayne State University in the United States and Guangxi CDC in China reviewed and approved the research protocol and the consent procedure.

### Measurements

#### Demographics

Demographic information included participants’ gender (male vs. female), age (in years), years of school (in years), ethnicity (Han, Zhuang, or others), religious beliefs (e.g., no-religious, Buddhism, and others), marital status (e.g., never vs. ever married), job categories (e.g., peasant workers, service staff, self-employed, unemployed, and others), residence registration (a.k.a. Hukou, urban vs. rural), residence types of the current place (urban vs. rural) and monthly income (in Chinese currency *Yuan*). Following the guideline from the Department of Health & Human Service (DHHS) [6], we used educational attainment, income levels, job category and residence type as indicators for PLWHA’s SES. People’s education attainment was measured by their reported “years of school”. Two groups (e.g., at least 9 years, and more than 9 years) based upon China’s Compulsory Education Law were generated [28]. PLWHA’s monthly income levels were further categorized into four groups based upon data distribution (e.g. “≤999”, “1000–1999”, “2000–2999”, and “≥3000”).

#### Exposure variables

We employed the validated Berger HIV Stigma Scale to measure three types of stigma: perceived, internalized, and enacted stigma [17,29,30]. Each stigma item was measured by a Likert-type scale (e.g., strongly disagree, disagree, agree, strongly agree) with a higher values indicating a greater agreement with the statement. Perceived stigma (α = 0.91) was measured by a few questions related to awareness of societal norms and prejudicial actions towards PLWHA (e.g., “most people consider PLWHA filthy”). Internalized stigma (α = 0.92) was evaluated by their negative feelings about oneself because of contracting with HIV (e.g., I feel guilty because I have HIV). Enacted stigma (α = 0.63) was measured by questions regarding discriminatory experience that the PLWHA have encountered (e.g., I will lose my job if my sero-status is known by others). The overall Cronbach’s α was 0.927 for the 16-item stigma scale in the current study. For the purpose of data analysis, we divided the total stigma score by its quartiles in order to assess if there were any trends of outcome variables with the increased stigma scores. Outcome variables

Indicators for psychosocial well-being included assessments of depression (Shorten version of Center for Epidemiological Studies Depression Scale for Children [CESD-10] with 10 item; α = 0.76), anxiety (Zung Self-Rating Anxiety Scale [SAS] with 20 items; α = 0.91), HIV management related self-esteem (α = 0.94), resilience (The 10-item Connor-Davidson Resilience Scale with 10 items; α = 0.96), social support (The Multidimensional Scale of Perceived Social Support [MSPSS], and The Medical Outcomes Study Social Support Survey [MOS-SSS-C] with 28 items; α = 0.98), and coping skills (Family Coping Project Coping Scale [FCPCS] with 25 items; α = 0.93). These assessment scales have been validated in previous studies conducted in Chinese settings [31,32]. Substance use behaviors were measured by questions asking if participants ever had used tobacco, drug or alcohol (yes vs. no) in the past six months. If participants answered “yes” to any of the questions, they would be coded as “ever”, otherwise, “never” was coded.

### Analytical plan

Several analytic procedures were employed to assess how stigma affected psychosocial well-being and substance use behaviors. First, we compared the demographic characteristics, psychosocial well-being indicators and substance use behaviors using ANOVA (for continuous variables) and Chi-squares tests (for categorical variables) among PLWHA at different SES gradients. Second, we explored how different types of HIV stigma impacted psychosocial well-being and substance use behaviors using multivariate regression analyses while adjusting for association-specific confounders. In addition, stratified analyses by different SES gradients (e.g., years of education, job category, residence type and monthly income) were further conducted. All data analyses were conducted using Stata 12.0® (StataCorp LP, College Station, Texas, USA).

## Results

### Characteristics of participants

In the current sample, majority of participants were of Han-ethnicity (70.8%), married (66.5%), without a religious belief (92.2%), having rural residence registration (84.4%). Of these participants, 2391 (80.2%) living in rural areas, 1748(58.5%) working as peasants, 2,584 (86.5%) having at most nine-year education, and more than half (53.2%) having monthly income less than 1,000 yuan (one USD = 6 yuan at the time of survey) (Table 1).

**Table 1:** Demographic information among PLWHA by SES indicators.

		Years of school (n = 2987)		Monthly Income (yuan) (n = 2958)				Residency of living (n = 2982)		Job category (n = 2987)				
DEMOGRAPHICS	Overall	< = 9yrs (n = 2584)	>9yr(n = 403)	≤999(n = 1572)	1000-1999(n = 870)	2000-2999(n = 334)	> = 3000(n = 182)	Urban(n = 591)	Rural(n = 2391)	Peasant workers(n = 1748)	Services(n = 138)	Self-employed(n = 342)	Unemployed(n = 406)	Others(n = 353)
**Ethnicity (%)**														
Han	70.76	70.8	70.22	70.92	69.04	72.75	73.63*	72.59	70.27	68.77	68.61	76.61	73.33	72.52
Zhuang	25.88	25.79	26.8	26.79	25.89	23.05	24.18	24.7	26.26	28.14	27.01	20.76	22.47	23.51
Others	3.36	3.41	2.98	2.3	5.06	4.19	2.2	2.71	3.48	3.09	4.38	2.63	4.2	3.97
**Religious (%)**														
No	92.24	92.54	90.5	90.16	93.75	96.71	95.53****	95.57	91.47**	90.35	98.55	93.55	94.53	95.4****
Yes	7.76	7.46	9.5	9.84	6.25	3.29	4.47	4.43	8.53	9.65	1.45	6.45	5.47	4.6
**Hukou (Residence Registration)**														
Urban	15.56	11.16	43.92****	11.72	17.38	24.25	23.08****	74.96	0.92****	3.15	29.71	28.65	31.03	41.08****
Rural	84.44	88.84	56.08	88.28	82.62	75.75	76.92	25.04	99.08	96.85	70.29	71.35	68.97	58.92
**Marital status**														
Married	66.45	66.38	67.09	62.39	71.73	70.91	70.06****	60.93	67.85**	69.88	56.3	63.99	52.53	72.05****
Not married	33.55	33.62	32.91	37.61	28.27	29.09	29.94	39.07	32.15	30.12	43.7	36.01	47.47	27.95

Notes:

*p<0.05

**p<0.01

***p<0.001

****p<0.0001

### Psychosocial wellbeing among PLWHA at different SES gradients

There were significant differences by SES gradients for most indicators of psychosocial well-being and substance use behaviors. Specifically, PLWHA with longer years of school, higher income levels, living at urban areas and working as “others” showed significantly better psychosocial wellbeing, less drug use behaviors, and less perceived and internalized stigma (*p*<0.05) (Table 2).

**Table 2:** Psychosocial well-being and substance use among PLWHA by SES indicators.

		Year of school (n = 2987)		Income levels (n = 2958)				Residency of living (n = 2982)		Job category (n = 2987)				
	Overall	< = 9yrs (n = 2584)	>9yr(n = 403)	≤999(n = 1572)	1000-1999(n = 870)	2000-2999(n = 334)	> = 3000(n = 182)	Urban(n = 591)	Rural(n = 2391)	Peasant workers	Services	self-employed	Unemployed	Others
**Depression (mean, SD)**	7.78(4.90)***	7.74(4.91)	8.06(4.79)	8.08(5.20)	7.56(4.57)	7.06(4.32)	7.17(4.22) ****	7.98(4.56)	7.73(4.97)	7.63(4.92)	7.24 (4.20)	7.58 (4.52)	9.58 (5.48)	6.85(4.13) ****
**Anxiety**	31.07 (8.85) **	31.04 (8.81)	31.35(9.23)	32.13(9.48)	30.13(8.21)	29.04 (7.16)	29.69 (7.56) ****	30.68(8.79)	31.18 (8.89)	31.03 (8.59)	29.30 (6.68)	30.57 (7.89)	34.55 (11.26)	28.55(7.43) ****
**Resilience**	3.19(0.84)**	3.15 (0.83)	3.43(0.86) ****	3.06(0.85)	3.26(0.81)	3.46(0.81)	3.47(0.79) ****	3.38(0.88)	3.15(0.83) ****	3.13(0.84)	3.39(0.81)	3.32(0.82)	3.07 (0.87)	3.42(0.79) ****
**Coping**	2.53(0.65)**	2.51 (0.65)	2.66(0.64) ****	2.48(0.64)	2.53(0.61)	2.62(0.67)	2.76(0.75) ****	2.62(0.69)	2.51(0.64) ****	2.48(0.64)	2.59(0.66)	2.57(0.60)	2.60 (0.69)	2.62(0.67)***
**Self-esteem**	3.32(0.72) ***	3.30 (0.70)	3.39(0.83)*	3.21(0.73)	3.36(0.69)	3.60(0.65)	3.52(0.69) ****	3.35(0.81)	3.31(0.69)	3.29(0.69)	3.45(0.69)	3.42(0.70)	3.11 (0.82)	3.53(0.70) ****
**Social Support**	2.42(0.85)*	2.38 (0.83)	2.68(0.93) ****	2.34(0.82)	2.41(0.84)	2.64(0.89)	2.77(0.94) ****	2.57(0.93)	2.38(0.82) ****	2.34(0.81)	2.48(0.93)	2.58(0.87)	2.47 (0.87)	2.58(0.90) ****
**Smoking (%)**	51.57****	51.48	52.13	52.53	50.40	48.95	54.44	49.75	52.04	50.49	32.12	50.44	60.40	55.40 ****
**Drug use (%)**	19.35****	20.40	12.50****	24.14	13.99	13.51	14.36****	18.47	19.50	16.11	7.97	22.51	39.60	13.35 ****
**Alcohol drinking (%)**	42.76	41.37	52.62****	39.46	45.39	50.90	46.96****	48.81	41.46 **	39.94	41.61	49.41	45.19	49.00 **
**Perceived stigma**	15.54 (3.53) **	15.63 (3.43)	14.90(4.07) ***	15.72(3.62)	15.45(3.39)	14.93 (3.42)	15.46 (3.39)*	14.96(4.11)	15.67(3.36)****	15.63 (3.41)	15.67 (3.64)	15.20 (3.67)	15.60 (3.59)	15.24 (3.85)
**Internalized stigma**	18.50 (4.35) ***	18.60 (4.27)	17.80(4.81) ***	18.72(4.45)	18.26(4.16)	17.88 (4.28)	18.74 (4.45)*	17.70(4.86)	18.69(4.20)****	18.61 (4.25)	18.38 (4.63)	18.03 (4.49)	18.76 (4.48)	18.06 (4.44)*
**Enacted stigma**	2.09(0.46)	2.08 (0.43)	2.16(0.57) **	2.11(0.49)	2.09(0.42)	2.05(0.36)	2.09(0.44)	2.10(0.45)	2.09(0.46)	2.09(0.44)	2.07(0.33)	2.08(0.44)	2.15 (0.59)	2.08(0.38)

Notes: SD: standard deviation

*p<0.05

** p<0.01

***p<0.001

****p<0.0001

### Impacts of HIV stigma

The overall multivariate analyses revealed that each type of HIV stigma is positively associated with psychosocial distress (e.g., depression and anxiety), but negatively related to protective buffers (e.g., resilience and self-esteem) of PLWHA across all SES gradients with enacted stigma having the strongest impact. For instance, compared to perceived (β = 0.20, 95% CI = 0.11, 0.28)) and internalized stigma (β = 0.55, 95% CI = 0.49, 0.62), the magnitudes of having anxiety problems was much higher among PLWHA who reported enacted stigma (β = 3.80, 95% CI = 3.18, 4.41). Similarly, the odds of smoking (aOR = 1.35, 95% CI = 1.04, 1.76) were significantly higher among PLWHA encountering enacted stigma compared to these who reported other stigma types (for perceived stigma: aOR = 0.99 [95% CI = 0.96, 1.02], for internalized stigma: aOR = 1.00 [aOR = 0.97, 1.02]). The same pattern has been observed for other indicators of psychosocial wellbeing and behaviors of substance use among PLWHA (Table 3).

**Table 3:** Associations between different types of stigma and psychosocial well-being as well as substance use by education level (N = 2987).

	Stigma	Overall	Years of school < = 9yrs	Years of school >9yrs
**Psychosocial Well-being**		**Adjusted β (95%CI)**	**Adjusted β (95%CI)**	**Adjusted β (95%CI)**
**Anxiety**^**a**^	**Enacted**	3.80(3.18,4.41)****	3.74(3.04,4.44)****	3.37(2.05,4.68)****
	**Perceived**	0.20(0.11,0.28)****	0.16(0.07,0.26)**	0.36(0.16,0.55)****
	**Internalize**	0.55(0.49,0.62)****	0.53(0.46,0.61)****	0.66(0.50,0.82)****
**Depression**^**b**^	**Enacted**	1.64(1.28,1.99)****	1.58(1.17,1.99) ****	1.64(0.89,2.38) ****
	**Perceived**	0.16(0.12,0.21)****	0.16(0.11,0.21) ****	0.18(0.07,0.29) **
	**Internalize**	0.26(0.23,0.30)****	0.26(0.22,0.30) ****	0.28(0.19,0.37) ****
**Resilience** ^**c**^	**Enacted**	-0.16(-0.22,-0.09)****	-0.15(-0.22,-0.08) ****	-0.19(-0.34,-0.05) *
	**Perceived**	-0.03(-0.04,-0.02)****	-0.02(-0.03,-0.01) ****	-0.05(-0.07,-0.03) ****
	**Internalize**	-0.04(-0.05,-0.04)****	-0.04(-0.05,-0.03) ****	-0.07(-0.09,-0.05) ****
**Self-esteem**^**d**^	**Enacted**	-0.19(-0.24,-0.14)****	-0.18(-0.24,-0.12) ****	-0.19(-0.32,-0.05) **
	**Perceived**	-0.03(-0.03,-0.02)****	-0.02(-0.03,-0.02) ****	-0.04(-0.06,-0.02) ****
	**Internalize**	-0.02(-0.03,-0.02)****	-0.02(-0.02,-0.01)****	-0.06(-0.07,-0.04) ****
**Coping** ^**e**^	**Enacted**	0.01(-0.04,0.06)	0.00(-0.06,0.06)	0.06(-0.06,0.17)
	**Perceived**	0.01(0.01,0.02)****	0.01(0.00,0.02)**	0.02(0.00,0.03)
	**Internalize**	0.02(0.01,0.02)****	0.02(0.01,0.02)****	0.01(0.00,0.03)
**Social Support**^**f**^	**Enacted**	-0.11(-0.17,-0.04)*	-0.16(-0.23,-0.08)****	0.08(-0.08,0.24)
	**Perceived**	-0.02(-0.03,-0.01)****	-0.01(-0.02,-0.01)**	-0.03(-0.05,0.00)*
	**Internalize**	-0.02(-0.03,-0.02)****	-0.02(-0.03,-0.01)****	-0.05(-0.07,-0.03)****
**Substance Use**	**Stigma**	**Adjusted OR (95%CI)**	**Adjusted OR (95%CI)**	**Adjusted OR (95%CI)**
**Smoking**^**g**^	**Enacted**	1.35(1.04,1.76)*	1.35(1.01,1.82)*	1.35(0.73,2.50)
	**Perceived**	0.99(0.96,1.02)	0.99(0.96,1.02)	1.03(0.96,1.12)
	**Internalize**	1.00(0.97,1.02)	0.99(0.97,1.02)	1.00(0.94,1.07)
**Drug** ^**h**^	**Enacted**	1.12(0.91,1.40)	1.07(0.83,1.38)	1.27(0.82,1.97)
	**Perceived**	1.06(1.02,1.10)**	1.06(1.02,1.10)**	1.12(1.01,1.24)*
	**Internalize**	1.01(0.98,1.04)	1.00(0.97,1.03)	1.06(0.98,1.15)
**Alcohol** ^**i**^	**Enacted**	0.97(0.81,1.17)	0.99(0.81,1.22)	0.98(0.66,1.45)
	**Perceived**	0.97(0.94,0.99)**	0.97(0.94,1.00)*	0.97(0.91,1.02)
	**Internalize**	0.97(0.95,0.99)**	0.97(0.95,0.99)**	0.97(0.92,1.02)

Notes:

Model _a-f_: adjusted for demographics, substance use and physical fitness

Model _g-i_: adjusted for demographics and physical fitness

*p<0.05

** p<0.01

****p<0.0001

When we examined the HIV stigma by SES-gradients, our findings indicated that impact of HIV stigma varied by different indicators of SES. PLWHA with longer years of schooling, stigma usually had worse impact on their psychosocial wellbeing status and problems of substance use, while for people with higher income, the impact of the least compared to their peers with lower incomes. For instance, for PLWHA who had shorter years of schooling, the impact of stigma on depression, resilience, coping and social support was at a lower degree compared to people with longer years of schooling, while the impact was stronger for coping and social support among PLWHA with less school education. For the impact on substance use, enacted stigma increased the odds of smoking (aOR = 1.35, 95% CI = 1.01, 1.82) among PLWHA with short years of schooling, but the odds of alcohol use got decreased for PLWHA experiencing perceived (aOR = 0.97, 95% CI = 0.94, 1.00) and internalized stigma (aOR = 0.97, 95% CI = 0.95, 0.99) (Table 3).

For PLWHA with more than 3000 yuan monthly income, the impact of stigma on most indicators for psychosocial wellbeing was insignificant. However, among PLWHA with highest income, enacted stigma increased the odds of smoking (aOR = 5.78, 95% CI = 1.77, 18.85) and alcohol use (aOR = 2.40, 95% CI = 1.03, 5.60); internalized stigma increased the odds of drug use (aOR = 1.16, 95% CI = 1.02, 1.33). For PLWHA with less than 999 yuan monthly income, the impact of stigma on psychosocial status was strongest in terms of the magnitude of the odds compared to their high-income peers (Table 4).

**Table 4:** Associations between different types of stigma and psychosocial well-being as well as substance use by income levels (N = 2987).

	Stigma	≤999	1000–1999	2000–2999	> = 3000
**Psychosocial Well-being**		**Adjusted β (95%CI)**	**Adjusted β (95%CI)**	**Adjusted β (95%CI)**	**Adjusted β (95%CI)**
**Anxiety**^**a**^	**Enacted**	4.08(3.26,4.91)****	3.87(2.66,5.09)****	1.42(-0.52,3.36)	2.19(-0.12,4.51)
	**Perceived**	0.22(0.10,0.34)****	0.11(-0.04,0.27)	0.19(-0.02,0.40)	0.32(0.05,0.60)*
	**Internalize**	0.59(0.50,0.69) ****	0.50(0.38,0.62) ****	0.42(0.26,0.59) ****	0.50(0.29,0.71) ****
**Depression**^**b**^	**Enacted**	1.88(1.41,2.36)****	1.34(0.63,2.05)****	0.82(-0.39,2.04)	0.39(-1.05,1.83)
	**Perceived**	0.20(0.13,0.26) ****	0.16(0.07,0.25) ****	0.09(-0.04,0.22)	-0.01(-0.19,0.16)
	**Internalize**	0.30(0.24,0.35) ****	0.28(0.21,0.35) ****	0.17(0.06,0.27) ***	0.08(-0.06,0.22)
**Resilience** ^**c**^	**Enacted**	-0.19(-0.27,-0.10) ****	-0.16(-0.29,-0.03)*	0.07(-0.17,0.30)	-0.23(-0.51,0.05)
	**Perceived**	-0.02(-0.04,-0.01) ****	-0.04(-0.05,-0.02) ****	-0.03(-0.05,0.00)*	0.00(-0.03,0.03)
	**Internalize**	-0.04(-0.05,-0.03) ****	-0.05(-0.07,-0.04) ****	-0.04(-0.06,-0.02) ****	-0.01(-0.04,0.01)
**Self-esteem**^**d**^	**Enacted**	-0.20(-0.27,-0.13) ****	-0.21(-0.32,-0.10) ****	-0.08(-0.27,0.11)	-0.27(-0.52,-0.02)*
	**Perceived**	-0.03(-0.04,-0.02) ****	-0.03(-0.04,-0.02) ****	-0.04(-0.06,-0.02) ****	0.01(-0.02,0.04)
	**Internalize**	-0.02(-0.03,-0.02) ****	-0.03(-0.05,-0.02) ****	-0.02(-0.04,0.00) *	0.00(-0.02,0.03)
**Coping** ^**e**^	**Enacted**	0.03(-0.03,0.09)	-0.06(-0.16,0.04)	0.08(-0.13,0.28)	0.04(-0.25,0.33)
	**Perceived**	0.02(0.01,0.02)**	0.01(-0.01,0.02)	0.01(-0.02,0.03)	0.04(0.00,0.07*
	**Internalize**	0.01(0.01,0.02)**	0.01(0.00,0.02)	0.02(0.01,0.04)	0.04(0.02,0.07*
**Social Support**^**f**^	**Enacted**	-0.12(-0.20,-0.04)**	-0.14(-0.28,0.00)*	0.02(-0.26,0.29)	-0.01(-0.35,0.34)
	**Perceived**	-0.01(-0.02,0.00)*	-0.02(-0.04,0.00)*	-0.04(-0.07,-0.01)*	0.00(-0.04,0.04)
	**Internalize**	-0.03(-0.04,-0.02) ****	-0.03(-0.04,-0.01) ****	-0.01(-0.04,0.01)	-0.01(-0.04,0.01)
**Substance Use Stigma**		**aOR (95%CI)**	**aOR (95%CI)**	**aOR (95%CI)**	**aOR (95%CI)**
**Smoking**^**g**^	**Enacted**	1.37(0.98,1.91)	1.26(0.71,2.22)	0.53(0.19,1.47)	5.78(1.77,18.85)**
	**Perceived**	0.98(0.94,1.03)	1.00(0.94,1.06)	1.06(0.97,1.15)	0.96(0.83,1.12)
	**Internalize**	0.95(0.92,0.99)*	1.04(0.99,1.09)	1.08(1.00,1.16)*	0.99(0.89,1.11)
**Drug** ^**h**^	**Enacted**	1.19(0.92,1.55)	0.93(0.57,1.51)	1.13(0.39,3.24)	n/a (n/a, n/a)
	**Perceived**	1.05(1.00,1.10)*	1.08(1.00,1.17)*	1.24(1.06,1.45)**	1.08(0.90,1.28)
	**Internalize**	0.99(0.96,1.03)	1.02(0.96,1.08)	1.05(0.96,1.16)	1.16(1.02,1.33)*
**Alcohol** ^**i**^	**Enacted**	0.85(0.67,1.07)	1.26(0.84,1.89)	0.61(0.26,1.44)	2.40(1.03,5.60)*
	**Perceived**	0.94(0.91,0.98)**	1.00(0.96,1.05)	0.98(0.91,1.06)	0.99(0.89,1.10)
	**Internalize**	0.96(0.93,0.98)**	0.99(0.95,1.02)	1.01(0.95,1.07)	0.97(0.90,1.05)

Notes:

Model _a-f_: adjusted for demographics, substance use

Model _g-i_: adjusted for demographics

*p<0.05

** p<0.01

***p<0.001

****p<0.0001

For PLWHA living in rural areas, enacted stigma had stronger impact on their depression (β = 1.68, 95% CI = 1.28,2.08), resilience (β = -0.15, 95% CI = -0.22, -0.08), self-esteem (β = -0.20, 95% CI = -0.26,-0.14) and social support (β = -0.11, 95% CI = -0.18, -0.04) compared to people living in urban areas, but not for any substance use behaviors (p>0.05). On the other hand, perceived stigma increased the coefficients at a greater magnitude for PLWHA living in urban areas on their anxiety (β = 0.32, 95% CI = 0.17, 0.48), depression (β = 0.26, 95%CI = 0.17, 0.34) and coping skills (β = 0.02, 95% CI = 0.00, 0.03) compared with their peers living in rural areas. Only internalized stigma decreased the odds of alcohol use among PLWHA living in both urban (aOR = 0.96, 95%CI = 0.93, 1.00) and rural areas (aOR = 0.97, 95%CI = 0.95, 0.99). Perceived stigma increased the odds of drug use (aOR = 1.06, 95% CI = 1.02, 1.11), but decreased the odds of alcohol use (aOR = 0.97, 95%CI = 0.94, 0.99) among PLWHA living in rural areas (Table 5).

**Table 5:** Associations between different types of stigma and psychosocial well-being as well as substance use by residence status (rural vs. urban) (N = 2987).

	Stigma	Urban	Rural
**Psychosocial Well-being**		**Adjusted β (95%CI)**	**Adjusted β (95%CI)**
**Anxiety**^**a**^	**Enacted**	4.90(3.55,6.25) ****	3.51(2.82,4.21) ****
	**Perceived**	0.32(0.17,0.48) ****	0.16(0.06,0.26) **
	**Internalize**	0.49(0.36,0.62) ****	0.58(0.50,0.66) ****
**Depression**^**b**^	**Enacted**	1.58(0.81,2.35) ****	1.68(1.28,2.08) ****
	**Perceived**	0.26(0.17,0.34) ****	0.13(0.08,0.19) ****
	**Internalize**	0.32(0.25,0.39) ****	0.25(0.20,0.29) ****
**Resilience** ^**c**^	**Enacted**	-0.11(-0.27,0.04)	-0.15(-0.22,-0.08) ****
	**Perceived**	-0.02(-0.04,0.00)*	-0.03(-0.04,-0.02) ****
	**Internalize**	-0.05(-0.06,-0.03) ****	-0.04(-0.05,-0.04) ****
**Self-esteem**^**d**^	**Enacted**	-0.13(-0.27,0.01)	-0.20(-0.26,-0.14) ****
	**Perceived**	-0.03(-0.04,-0.01)**	-0.03(-0.03,-0.02) ****
	**Internalize**	-0.03(-0.05,-0.02) ****	-0.02(-0.03,-0.02) ****
**Coping** ^**e**^	**Enacted**	0.06(-0.07,0.18)	0.01(-0.05,0.07)
	**Perceived**	0.02(0.00,0.03)*	0.01(0.00,0.02)**
	**Internalize**	0.02(0.00,0.03)*	0.02(0.01,0.02) ****
**Social Support**^**f**^	**Enacted**	-0.04(-0.21,0.13)	-0.11(-0.18,-0.04) **
	**Perceived**	-0.02(-0.04,0.00)	-0.02(-0.03,-0.01) **
	**Internalize**	-0.03(-0.05,-0.02) ****	-0.02(-0.03,-0.01) ****
**Substance Use**	**Stigma**	**Adjusted OR (95%CI)**	**Adjusted OR (95%CI)**
**Smoking**^**g**^	**Enacted**	1.49(0.90,2.46)	1.26(0.92,1.72)
	**Perceived**	1.01(0.95,1.06)	0.98(0.95,1.02)
	**Internalize**	1.02(0.98,1.07)	0.99(0.96,1.02)
**Drug** ^**h**^	**Enacted**	0.94(0.59,1.52)	1.10(0.86,1.41)
	**Perceived**	1.05(0.98,1.12)	1.06(1.02,1.11)**
	**Internalize**	1.04(0.98,1.09)	1.00(0.96,1.03)
**Alcohol** ^**i**^	**Enacted**	0.84(0.56,1.26)	0.99(0.81,1.21)
	**Perceived**	0.97(0.93,1.01)	0.97(0.94,0.99)**
	**Internalize**	0.96(0.93,1.00)*	0.97(0.95,0.99)*

Notes:

Model _a-f_: adjusted for demographics, substance use

Model _g-i_: adjusted for demographics

*p<0.05

** p<0.01

***p<0.001

****p<0.0001

For PLWHA working as unemployed or peasant workers, their psychosocial status was most impacted by HIV stigma. Among unemployed PLWHA, the internalized stigma impacted the odds of anxiety (β = 0.65, 96%CI = 0.43, 0.87), depression (β = 0.33, 96%CI = 0.22, 0.44), resilience (β = -0.06, 96%CI = -0.08,-0.04), coping (β = 0.03, 96%CI = 0.01, 0.04), and social support (β = -0.03, 96%CI = -0.05,-0.01) to the greatest magnitude. While for PLWHA working as peasant workers, their drug use behavior (aOR = 1.06, 95%CI = 1.01, 1.12) was impacted significantly by their encountered perceived stigma, and their alcohol use was negatively affected by their experienced perceived (aOR = 0.96, 95% CI = 0.93, 0.99) and internalized stigma (aOR = 0.97, 95% CI = 0.94, 1.00) (Table 6).

**Table 6:** Associations between different types of stigma and psychosocial well-being as well as substance use by occupational type (N = 2987).

	Stigma	Peasant workers	Services	Self-employed	Unemployed	Others
**Psychosocial Well-being**		**Adjusted β (95%CI)**	**Adjusted β (95%CI)**	**Adjusted β (95%CI)**	**Adjusted β (95%CI)**	**Adjusted β (95%CI)**
**Anxiety**^**a**^	**Enacted**	3.42(2.61,4.23)****	3.55(0.58,6.52)*	4.10(2.34,5.86)****	4.03(2.38,5.68)****	4.23(2.38,6.09)****
	**Perceived**	0.18(0.07,0.29)**	-0.02(-0.33,0.29)	0.30(0.08,0.52)**	0.17(-0.12,0.46)	0.24(0.06,0.43)*
	**Internalize**	0.56(0.47,0.64) ****	0.45(0.22,0.68) ****	0.48(0.31,0.66) ****	0.65(0.43,0.87) ****	0.50(0.35,0.66) ****
**Depression**^**b**^	**Enacted**	1.25(0.76,1.74) ****	0.22(-1.67,2.11)	2.89(1.83,3.96) ****	1.90(1.07,2.73) ****	1.23(0.08,2.38)*
	**Perceived**	0.16(0.09,0.22) ****	-0.06(-0.26,0.13)	0.23(0.09,0.36) **	0.15(0.00,0.29)*	0.20(0.09,0.31) ****
	**Internalize**	0.25(0.20,0.30) ****	0.12(-0.03,0.27)	0.31(0.20,0.42) ****	0.33(0.22,0.44) ****	0.29(0.19,0.38) ****
**Resilience** ^**c**^	**Enacted**	-0.20(-0.29,-0.12) ****	-0.35(-0.71,0.02)	-0.07(-0.26,0.11)	-0.10(-0.24,0.04)	-0.13(-0.35,0.10)
	**Perceived**	-0.03(-0.04,-0.02) ****	-0.03(-0.07,0.01)	-0.02(-0.05,0.00)*	-0.03(-0.06,-0.01)*	-0.02(-0.05,0.00)*
	**Internalize**	-0.04(-0.05,-0.03) ****	-0.05(-0.07,-0.02) **	-0.04(-0.06,-0.02) ****	-0.06(-0.08,-0.04) ****	-0.03(-0.05,-0.01)**
**Self-esteem**^**d**^	**Enacted**	-0.22(-0.29,-0.15) ****	-0.12(-0.45,0.20)	-0.05(-0.21,0.12)	-0.19(-0.32,-0.06)**	-0.17(-0.36,0.02)
	**Perceived**	-0.03(-0.04,-0.02) ****	-0.04(-0.08,-0.01)**	-0.03(-0.05,-0.01)**	-0.01(-0.04,0.01)	-0.03(-0.04,-0.01)**
	**Internalize**	-0.03(-0.03,-0.02) ****	-0.04(-0.06,-0.01)**	-0.03(-0.04,-0.01)**	-0.02(-0.04,0.00)*	-0.03(-0.05,-0.01) ****
**Coping** ^**e**^	**Enacted**	-0.02(-0.09,0.05)	-0.07(-0.40,0.26)	0.04(-0.12,0.19)	0.07(-0.04,0.19)	0.07(-0.13,0.27)
	**Perceived**	0.01(0.00,0.02)	0.02(-0.01,0.05)	0.02(0.00,0.04)*	0.02(0.00,0.04)*	0.01(0.00,0.03)
	**Internalize**	0.01(0.00,0.02) **	0.03(0.01,0.06) **	0.03(0.02,0.05) ****	0.03(0.01,0.04) **	0.01(-0.01,0.03)
**Social Support**^**f**^	**Enacted**	-0.13(-0.22,-0.04)**	-0.23(-0.68,0.21)	-0.25(-0.47,-0.03)*	0.02(-0.12,0.17)	-0.14(-0.41,0.13)
	**Perceived**	-0.02(-0.03,0.00)**	-0.02(-0.07,0.02)	-0.01(-0.04,0.01)	-0.02(-0.04,0.01)	-0.02(-0.05,0.00)
	**Internalize**	-0.02(-0.03,-0.01) ****	-0.01(-0.04,0.03)	-0.02(-0.04,0.00)	-0.03(-0.05,-0.01) **	-0.03(-0.05,-0.01) **
**Substance Use Stigma**		**aOR (95%CI)**	**aOR (95%CI)**	**aOR (95%CI)**	**aOR (95%CI)**	**aOR (95%CI)**
**Smoking**^**g**^	**Enacted**	1.34(0.92,1.97)	2.69(0.68,10.64)	1.20(0.56,2.59)	1.76(0.89,3.50)	1.21(0.55,2.65)
	**Perceived**	0.99(0.95,1.03)	1.21(0.96,1.52)	1.04(0.94,1.14)	1.00(0.92,1.10)	0.93(0.86,1.00)*
	**Internalize**	0.98(0.95,1.02)	1.03(0.90,1.18)	1.04(0.96,1.12)	1.02(0.95,1.10)	0.99(0.93,1.05)
**Drug** ^**h**^	**Enacted**	1.11(0.82,1.51)	3.03(0.66,14.01)	1.16(0.58,2.31)	1.22(0.79,1.89)	1.46(0.67,3.20)
	**Perceived**	1.06(1.01,1.12)*	1.14(0.85,1.52)	1.09(0.98,1.20)	1.06(0.99,1.14)	1.05(0.94,1.17)
	**Internalize**	0.99(0.95,1.03)	1.05(0.86,1.27)	1.06(0.98,1.15)	1.02(0.96,1.08)	0.97(0.89,1.07)
**Alcohol** ^**i**^	**Enacted**	0.89(0.69,1.14)	1.89(0.64,5.61)	1.91(1.04,3.52)*	0.91(0.63,1.31)	1.07(0.57,2.01)
	**Perceived**	0.96(0.93,0.99)*	1.00(0.88,1.14)	0.94(0.87,1.01)	0.99(0.93,1.05)	0.99(0.93,1.06)
	**Internalize**	0.97(0.94,1.00)*	0.98(0.89,1.08)	0.95(0.89,1.00)	1.00(0.95,1.05)	0.98(0.93,1.04)

Notes:

Model _a-f_: adjusted for demographics, substance use

Model _g-i_: adjusted for demographics

*p<0.05

** p<0.01

***p<0.001

****p<0.0001

## Discussion

Our analyses revealed that stigma significantly impacted PLWHA’s psychosocial distress and behaviors of substance use, and the impacts varied by different SES gradients. Specifically, PLWHA with higher education may be more sensitive compared with these who had lower educational attainment. As a result, the magnitude of each type of stigma was higher in the more educated group. On the other hand, PLWHA with higher income may play as a buffer from suffering stigma, therefore, they were least impacted by HIV stigma. However, the enacted stigma had the highest odds on self-esteem, smoking and alcohol use among PLWHA with the highest level of monthly income. Perhaps PLWHA with higher income were more sensitive to their stigmatized experience (e.g., enacted stigma), but less likely to generate negative opinion towards themselves (e.g., internalized and perceived stigma). However, PLWHA with higher income were more likely to report higher likelihood of substance use than their peers with lower incomes. It is perhaps that PLWHA with higher income were more likely to afford their expense of substances compared to people with lower income.

Stratified analyses by the SES gradients provided a detailed profile for PLWHA with different SES were impacted by HIV stigma with various magnitudes, although the overall analyses can provide a general direction. For instance, the overall analyses revealed enacted stigma increased the odds of having anxiety problem among PLWHA by 3.8 times after controlling for potential confounders. Our stratified analyses indicated PLWHA with lower education and least income (e.g., <999 RMB/month) were impacted to the greatest extend by enacted stigma compared to their peers. Although the overall analyses revealed non-significant odds of drinking alcohol among PLWHA who encountered enacted stigma, our stratified analyses indicated that enacted stigma increased the odds of drinking alcohol for PLWHA with monthly income more than 3,000 RMB by 2.4 times, and increased the odds for PLWHA who were self-employed by 1.91 times. If no stratified analyses had been conducted, we may conclude that enacted stigma had least impact on PLWHA’s drinking behaviors. The differences between overall and stratified analyses can be observed throughout other indicators for psychosocial status and substance use in the current study. Such findings aimed to remind health professionals of having a pair of keen eyes to observe subtle differences among PLWHA with different SES backgrounds in order to design tailored interventions.

Consistent with existing studies, the psychosocial distress and substance use behaviors were more common among individuals with lower SES or living in lower-graded neighborhoods[9,33,34]. People having higher SES usually meant they possess more material (e.g., money, house), human (e.g., skills, knowledge), and social capital (e.g., social support, social network) compared to people with lower SES [5,35]. PLWHA with higher SES usually had lower mortality rate and better prognosis than their low-SES counterparts [3]. How the SES interacted with the association between HIV stigma and psychosocial problems was complex and required a more sophisticated study design to explore the mechanisms. Our study is the first paper to document the paucity of SES stratified analyses between HIV stigma and psychosocial status among PLWHA in China.

A few caveats should be acknowledged when interpreting findings in the current study. First, the nature of cross-sectional design constrained our capacity to make any causal inferences between HIV stigma and psychosocial status as well as substance use among PLWHA. We call for longitudinal studies in future research to examine any potential causality. Second, all data were collected via self-reporting, participants may underreport their substance use or psychosocial status due to social desirability bias. Therefore, misclassification of key variables may result in diluting the effect of stigma toward the null. Although audio computer-assisted self-interview techniques may increase the validity of collected data, the evidence was inconclusive as traditional face-to-face interview may not be uniformly inferior to non-interviewer techniques across all occasions [36]. Third, as the current study was conducted in a southwest region with many minorities, findings in the current study may be subject to limited generalizability to other settings in China. Fourth, due to the dynamic changes of the definition on SES, the way of measuring the SES among PLWHA in the current study may not be able to capture all domains of individual’s social, human and material capital as literatures suggested [5]. However, we followed the guideline suggested by the DHHS for practical and feasible purposes [6]. Last, due to limited space of the questionnaire, we did not collect detailed information on participants’ job categories. PLWHA who have “decent” jobs (e.g., government employees or school teachers) may encounter more HIV stigma compared to PLWHA working as peasant workers or service staff. In future studies, we suggest researchers employ hypotheses-driven strategies to explore how SES impact these studied associations.

To our knowledge, it is the first study to assess the stratified impacts of HIV stigma on PLWHA’s psychosocial status and behaviors of substance use by their SES gradients. Findings in the current study served as guideline for health professionals to design tailored interventions among PLWHA in future. For PLWHA with better education, living in urban areas and not working as peasant workers or unemployed, more interventions focusing on psychosocial health are needed. For PLWHA with higher income, more programs on improving coping strategies and reducing their substance use behaviors are desired. Only can we combine individual-level factors with social, historical and biophysical contexts of PLWHA, health professionals can better understand the disease etiology, health, and intervention modes for PLWHA in China.
